# Supplementary material for: The effects of genital myiasis on the diversity of the vaginal microbiota in female Bactrian camels
Source: BMC Vet Res. 2022 Mar 5;18:87. doi: 10.1186/s12917-022-03189-5 (PMC8897907; doi:10.1186/s12917-022-03189-5)
Supplement: Supplementary file 5 — Additional file 5. [file 12917_2022_3189_MOESM5_ESM.zip › MPL201709200_16s_yy/Treat1/css/js_css/pdfmake-master/examples/pdfs/textDecorations.pdf]

Higlighted text

Underline decoration Line Through decoration Overline decoration

Dashed style Dotted style Double style Wavy style

Using colors Using colors Using colors
